# Introduction of Triple-Drug Therapy for Accelerating Lymphatic Filariasis Elimination in India: Lessons Learned

**DOI:** 10.4269/ajtmh.21-0964

**Published:** 2022-03-15

**Authors:** Bhupendra Tripathi, Nupur Roy, Neeraj Dhingra

**Affiliations:** ^1^ Bill & Melinda Gates Foundation, Munirka, New Delhi, India;; ^2^ National Vector Borne Diseases Control Programme, New Delhi, India

## Abstract

There are 670 million people at risk of contracting lymphatic filariasis (LF) in India, which bears 40% of the global burden of the disease. The National Program to Eliminate LF was launched in 2004 first with a single-drug therapy—diethylcarbamazine (DEC), followed by a two-drug therapy—DEC + albendazole (DA). In 2017, following successful drug trials, World Health Organization endorsed a new triple-drug therapy to fight LF using ivermectin with DEC and albendazole (IDA). ^1^ In June 2018, India made new commitments to accelerate their program to eliminate LF and initiated the new IDA protocol in five districts in the country. This article looks at the experience of India in the roll out of the new drug protocol and shares their preparations, successes, challenges, and lessons learned.

## INTRODUCTION

Lymphatic filariasis (LF), or elephantiasis, is a mosquito-borne disease that disfigures and disables those who become infected. The disease was recorded in India as early as the sixth century BC by Sushruta in his book, *Sushruta Samhita*, and in the seventh century AD, Madhava-kara described signs and symptoms of the disease in his treatise, *Madhava Nidhana*. ^2^ In 1709, Clarke referred to elephantoid legs in Cochin (now Kochi) as “Malabar legs” and in 1872, microfilariae in the peripheral blood was discovered by Lewis in Kolkata city. ^2^ India bears around 40% of the global disease burden, with 272 LF endemic districts from 16 states and 5 union territories; close to 670 million people are at risk of contracting the disease. ^3^

India’s National Health Policy 2002 set a goal to eliminate LF as a public health problem in India by 2015, which was later extended to 2017. The goal set by the Global Program to Eliminate Lymphatic Filariasis was to eliminate LF by 2020. The global goal was subsequently revised to eliminate LF by 2030. National goals across endemic countries have aligned to meet that target.

## HISTORY OF MASS DRUG ADMINISTRATION FOR LF IN INDIA

**Figure 1.**
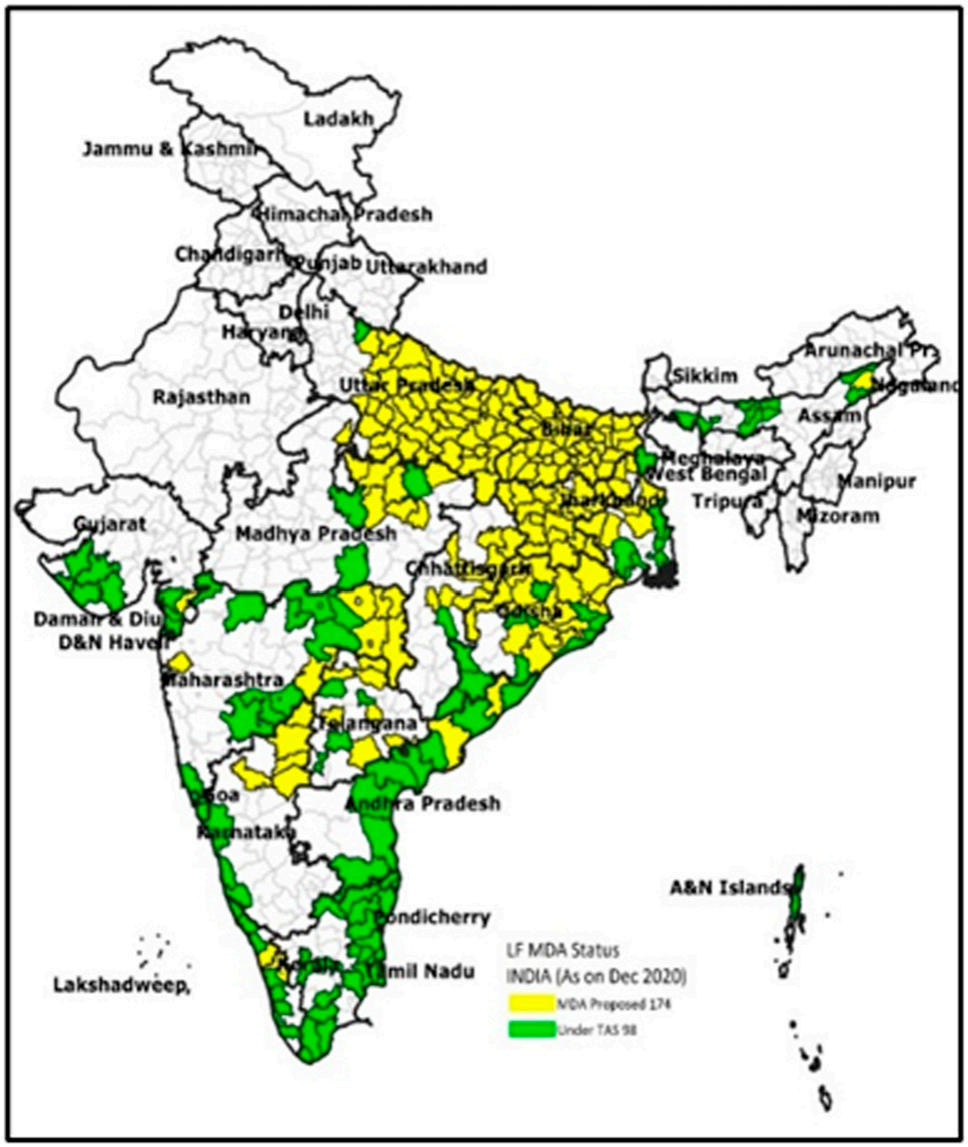
Lymphatic filariasis endemic districts of India, 2021. Source: National Vector Borne Diseases Control Program, Ministry of Health and Family Welfare, Government of India https://nvbdcp.gov.in/.

India launched the National Program to Eliminate Lymphatic Filariasis in 2004. As part of the program, an annual single dose of diethylcarbamazine (DEC) administered through mass drug administration (MDA) was introduced in 202 of the 256 endemic districts in India. By 2007, the MDA program had scaled up to cover 256 districts and included the administration of two drugs—DEC and albendazole (DA). In 2020, based on the remapping data, National Vector Borne Diseases Control Program (NVBDCP) added 16 new districts, making it a total of 272 LF endemic districts in the country. As of the start of 2021, 174 districts are still undergoing MDA, and 98 have stopped MDA and are undergoing various stages of transmission assessment surveys, which determine whether the transmission levels of LF are low enough to stop MDA (Figure 1).

Although the National Program to Eliminate Lymphatic Filariasis has made significant progress, India still has over 23 million people suffering with lifelong disability as a result of LF. Along with disability, this disease also inflicts stigma, mental suffering, and social deprivation, and is a major factor that perpetuates poverty in the affected communities. As of 2020, according to World Health Organization (WHO), ^4^ 430 million people in India continue to require annual preventive treatment through the MDA campaigns.

## IMPLEMENTATION CHALLENGES IN INDIA

Most of the 174 districts undergoing MDA (not including 16 newly added LF endemic districts) have implemented roughly 8–10 rounds of MDA in the last 15 years. In addition to poor community participation and low compliance, the program has faced multiple implementation challenges at a higher level. For years, the program suffered from a seeming impasse in which LF elimination was not a major part of the national public health agenda, few champions publicly voiced support for accelerating elimination efforts, little public discussion existed on the impact of LF on communities living in endemic regions, and national decision-making was slow in regard to adopting innovations to improve program efficiency. Frequent leadership transitions and postponement of technical advisory committee meetings also contributed to delays.

At the implementation level, delays in scheduling MDA rounds in endemic districts resulted in other management problems including delays in drug inventory projections, lack of availability of family registers for health staff, delays in development of district microplans, delays in training plans, and inaccurate counts of community members in urban and rural areas. The program also suffered from poor uptake of preventive drugs among community members during MDA due to deep-rooted systemic mistrust issues that were not addressed in a timely manner, leading to poor community participation and poor compliance with MDA. Limited external partner support in funding and technical assistance to help the states and districts successfully implement MDA further contributed to implementation challenges.

**Figure 2.**
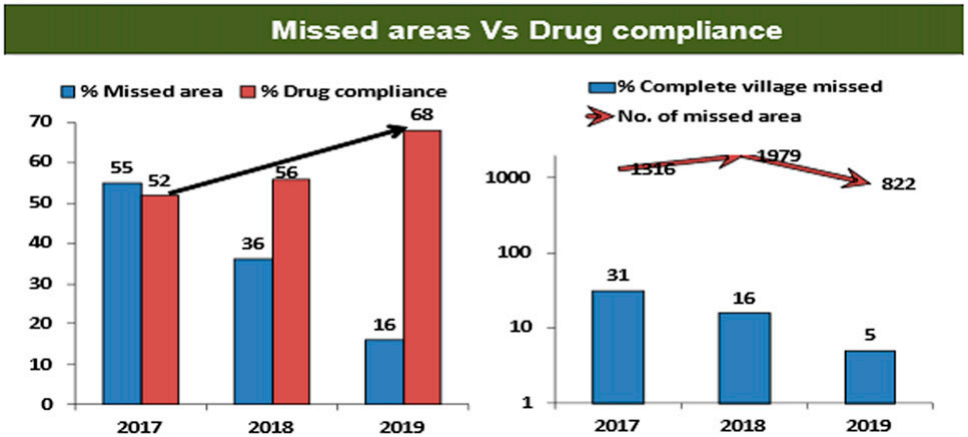
World Health Organization (WHO) field monitoring data. Source: WHO India Country Office LF MDA field monitoring data.

As a result of these challenges, the WHO country office for India expanded its technical support and shared strategies to address them through routine monitoring reports. The reports for 2017–2019 showed that over a period of 3 years, there was a significant decline in the number of “missed areas,” defined as areas where houses were missed and drugs were not offered. For the same time period, they showed a gradual improvement in the compliance among the population who received the drugs (Figure 2). These positive changes happened due to the strengthened efforts in advocacy, microplanning, training, monitoring, and social mobilization.

## GLOBAL IDA TRIAL RESULTS

Global trials in five countries including India between October 2016 and November 2017 showed that a single dose of triple-drug treatment—ivermectin with DEC and albendazole (IDA)—is superior to the double-drug combination of DEC and albendazole (DA). ^5^ In India, the IDA trial for LF was first carried out in selected blocks of Yadgir district in Karnataka state by the Indian Council of Medical Research’s Vector Control Research Center. Experts under the chairmanship of the director general of health services, Ministry of Health and Family Welfare, reviewed the outcomes of the trial. They found no serious adverse events and determined that IDA was more effective for clearing microfilariae than DA (84% versus 61.8%, *P* < 0.001). The expert group declared that IDA was a safe mass treatment regimen for LF elimination. ^6^

After assessing the results of the multicountry IDA trials, WHO updated its MDA guidelines by providing guidance to introduce IDA in selected geographies based on inclusion criterion. At the same time, the Mectizan Donation Program committed to providing 100 million treatments per year of ivermectin for IDA introduction globally until 2025.

## INTRODUCING IDA REGIMEN FOR LF IN INDIA

Renewing India’s commitment to accelerate LF elimination, the former Union Health Minister Shri. J. P. Nadda launched the Accelerated Plan for Elimination of Lymphatic Filariasis at the Global Alliance to Eliminate Lymphatic Filariasis meeting in June 2018 in New Delhi, India. The Ministry of Health and Family Welfare decided to introduce IDA initially in five districts of the country. The National Vector Borne Disease Control Program (NVBDCP) collaborated with partners—WHO, Bill & Melinda Gates Foundation, Project Concern International, PATH, and Global Health Strategies—to facilitate the rollout process at national, state, and district levels. ^7^

A technical committee was created under the chairmanship of the Directorate General of Health Services, and co-chaired by Dr. N. K. Ganguly, former Director General of Indian Council of Medical Research, with members from NVBDCP and WHO. The committee explored introduction of IDA in the LF MDA campaign in India. The committee was responsible for framing technical guidelines and policies, providing guidance for IDA implementation to states, overseeing adherence to safety protocols, and monitoring results of ground-level activities.

According to WHO recommendations, annual triple-drug IDA—rather than the double-drug combination—should be implemented in the following settings:

• Implementing units (administrative units such as blocks and districts) that have not yet started or have had fewer than four effective MDA rounds of DA.
• Implementing units that have not met appropriate epidemiological targets in sentinel and spot-check site surveys or that have failed transmission assessment surveys (or pre-Transmission Assessment Survey [TAS] surveys), despite meeting drug coverage targets during MDAs.
• Communities where post-MDA or postvalidation surveillance identified infection, which suggests local transmission.

With these recommendations in mind, the technical committee used the following criteria to identify districts for inclusion in the study: 1) districts that have failed transmission assessment surveys—two with predominantly rural areas and two with large urban areas; and 2) a district that has never undertaken MDA, but where LF transmission has been identified.

The technical committee selected Arwal district in Bihar and Simdega district in Jharkhand as the two small rural areas (Simdega had also not yet experienced MDA), and Nagpur district in Maharashtra and Varanasi district in Uttar Pradesh as the two large urban areas. The fifth district was Yadgir in Karnataka where IDA was previously administered as part of the global study for more than 6,000 people during the drug clinical trial. Whereas Simdega was to conduct MDA for the first time, the other four districts had failed transmission assessment surveys despite conducting more than five MDA rounds. IDA was rolled out in a staggered approach so that smaller areas could be targeted before moving on to larger areas. IDA implementation began in December 2018 and was completed in all five districts by the end of November 2019, thus making India the first country in Southeast Asia to roll out the innovative, WHO-approved triple-drug IDA regimen to accelerate LF elimination efforts.

The Government of India submitted a proposal to the Mectizan Donation Program, which works with ministries of health and partners by donating ivermectin for neglected tropical diseases through WHO. India’s proposal was to facilitate donation and delivery of ivermectin for close to 10.7 million people. Through WHO, the Mectizan Donation Program provided 31.27 million tablets of ivermectin during IDA rollout in the initial five districts. After successful introduction, the program further committed to providing 50 million doses of ivermectin for India annually until 2022 (Sharma P. News article: https://www.aninews.in/news/national/general-news/health-ministry-plans-to-introduce-mass-drug-administration-programme-in-yadgir-district-karnataka20190920165052/; Accessed July 13, 2021). Reported increases in drug compliance within communities encouraged national program managers to scale up innovations to accelerate LF elimination efforts. This paved the way for sustained scale-up of IDA in the country.

To ensure effective rollout of IDA, the Ministry of Health formed a team of experts from the Indian Council of Medical Research, NVBDCP, and WHO to develop new guidelines. The team formulated guidelines on how to administer doses of ivermectin using dose poles and other social mobilization innovations to enhance community compliance. Because ivermectin was to be given only to children age 5 and older or those taller than 90 cm, family registers and reporting formats were modified. Microplans were updated to include booths in schools as administration sites. Finger marking and house marking were introduced for door-to-door activities to help identify people who have received the MDA. Additional staff and volunteers were also deployed at the block level for training, social mobilization, monitoring, and review. Advocacy efforts were strengthened to include state health ministers, district government officials, and brand ambassadors to improve community acceptance and increase drug compliance for the IDA campaigns. IDA rollout in the initial five districts showed that the triple-drug IDA regimen was tolerated well, with no serious adverse events. The reported side effects were mild and comparable to the double-drug regimen.

WHO supported coverage evaluation surveys through local medical colleges for the initial four districts: Simdega, Arwal, Nagpur, and Varanasi as shown in Table 1. Data from the first round of IDA in these districts revealed that the predominantly rural districts had higher coverage (81%) than urban districts (39%). There was also a wide variation between the figures for administrative coverage data and the coverage evaluation survey compliance data.

**Table 1:** WHO CES results (IDA round 1 results from four pilot districts)

State	District	IDA use case	Population	Admin coverage (govt.)	Total			Rural			Urban		
					% Program reach*	% Program coverage†	% Compliance‡	% Program reach*	% Program coverage†	% Compliance‡	% Program reach*	% Program coverage†	% Compliance‡
Jharkand§	Simdega	Accelerate elimination	652,000	91	82	81	99	82	81	99	84	84	100
Bihar‖	Arwal	Validate elimination	770,000	83	66–70	58–61	87–88	Report did not provide rural/urban breakout					
Maharashtra¶	Nagpur	Validate elimination	2,872,000	85	67	54–55	81–82	80–82	73–74	90–91	49	42	86
UP^#^	Varanasi	Validate elimination	4,155,200	92	60–61	39–40	65	73–75	47–49	64–67	35–36	23–24	66–67

CES = coverage evaluation survey; IDA = ivermectin with DEC and albendazole.

Source: World Health Organization; Department of Community and Family Medicine, All India Institute of Medical Sciences; Department of Social and Preventative Medicine, Rajendra Institute of Medical Sciences, Ranchi, Jharkhand; Department of Community Medicine, Indira Gandhi Government Medical College, Nagpur; Institute of Medical Sciences, Banaras Hindu University, Varanasi.

* % Reach is the % of the total population reported to have been offered treatment.

† % Coverage is % of the total population reported to have consumed treatment.

‡ % Compliance is % of people reporting to have consumed the drugs of those offered the drugs.

§ Bihar—Conducted by Department of Community and Family Medicine, All India Institute of Medical Sciences (AIIMS) 6-month post-IDA. (IDA campaign Dec 2018; CVS June 2019).

‖ Jharkhand—Conducted by Department of Social and Preventive Medicine, Rajendra Institute of Medical Sciences, Ranchi, Jharkhand, 5-month post-IDA. Rural sample = 1,490 (93%); Urban sample = 111 (7%).

¶ Maharashtra—Conducted by Department of Community Medicine, Indira Gandhi Govt. Medical College, Nagpur.

# UP—Conducted by Institute of Medical Sciences, Banaras Hindu University, Varanasi. Rural sample = 1,281 (65%); Urban sample = 704 (35%).

## POST-IDA ASSESSMENT AND DECISION ON FURTHER SCALE-UP

The Ministry of Health in India decided that if IDA was tolerated well and found safe, it should be considered for scale-up in additional districts. In October 2019, WHO reported that 8.07 million of 10.7 million people had been treated during pilot rounds, a compliance rate of 75.4%, making a strong case for further scale-up of IDA in India. ^8^ NVBDCP organized a day-long national symposium on October 30, 2019, to accelerate India’s resolve toward LF elimination.

**Figure 3.**
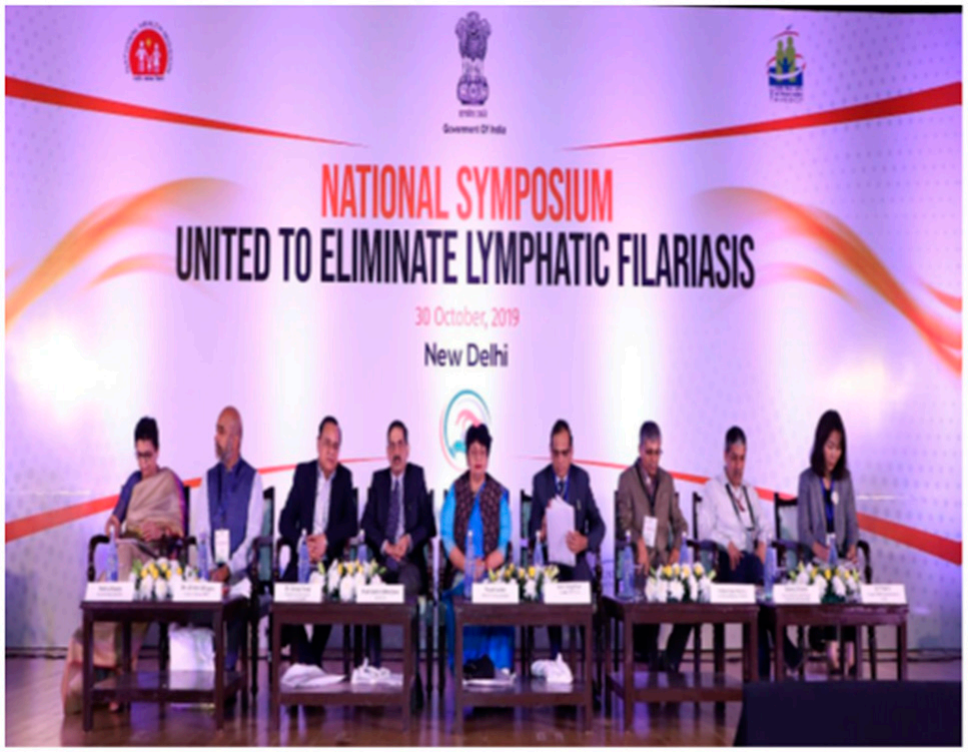
National lymphatic filariasis (LF) symposium 2018.

The national symposium, United to Eliminate Lymphatic Filariasis, brought over 300 participants to deliberate on building a common vision toward achieving LF elimination by 2021. Participants included global and national public health experts; representatives from 21 endemic states and union territories including principal secretaries from the LF high-burden states of Uttar Pradesh, Jharkhand, Maharashtra, and the director of the National Health Mission from Bihar; partners and donors; research organizations; and global and national pharmaceutical companies (Figure 3).

Dr. Harsh Vardhan, Union Health Minister, Ministry of Health and Family Welfare, announced an aggressive strategy to eliminate LF by 2021 with the national scale-up of triple-drug IDA therapy. He also recognized the states and districts who had successfully introduced IDA in the country and reiterated the prime minister’s vision for a healthy future for India.

## SCALE-UP OF IDA IN INDIA

With strong political will and administrative approvals, NVBDCP selected 21 additional LF endemic districts for IDA based on their MDA records, transmission assessment survey results (those who failed TAS despite high coverages), availability of drugs, and willingness of the states. Of the 21 newly identified districts, 17 were able to complete their first round of IDA by 2020. The second round of IDA in these districts were delayed due to the COVID-19 pandemic and is scheduled for the second half of 2021.

Among these 17 IDA scale-up districts, the WHO independent monitoring coverage data for the first round of IDA in 12 districts ranged from 25% to 84% as shown in Table 2 (in Hardoi district, due to a local administrative incident, health department went on strike, resulting in 25% coverage for the IDA round). Because of the COVID-19 lockdown, when house-to-house monitoring was not possible, five districts were not included in WHO’s coverage monitoring process during round 1.

**Table 2:** Round 1 coverage of triple-drug therapy in 17 districts

State	District	Round 1 coverage (%)	
		2019	2020
Maharashtra	Bhandara	–	House-to-house monitoring not done due to COVID-19 lockdown
	Chandrapur	–	House-to-house monitoring not done due to COVID-19 lockdown
	Gadchiroli	–	79.30
Uttar Pradesh	Allahabad	73.00	–
	Chandauli	54.00	–
	Fatehpur	69.00	–
	Hardoi	25.00	–
	Kanpur (Dehat)	55.00	–
	Kanpur (Nagar)	68.00	–
	Kheri	70.00	–
	Mirzapur	83.00	–
	Pratapgarh	63.00	–
	Sitapur	72.00	–
	Unnao	84.00	–
Gujarat	Tapi	–	House-to-house monitoring not done due to COVID-19 lockdown
Karnataka	Bidar	–	House-to-house monitoring not done due to COVID-19 lockdown
	Gulbarga	–	House-to-house monitoring not done due to COVID-19 lockdown

Source: WHO Independent Coverage Monitoring Data.

**Figure 4.**
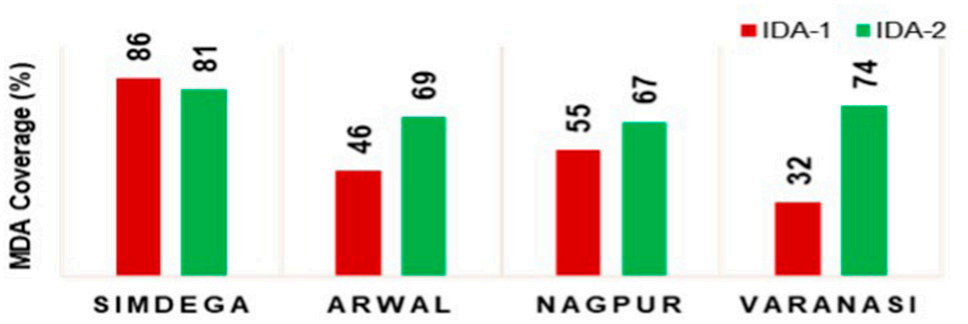
Comparison of ivermectin with DEC and albendazole (IDA) round 1 vs. IDA round 2 World Health Organization (WHO) coverage monitoring data.

In a comparison of IDA rounds 1 and 2 coverage data, we found a remarkable improvement in overall coverage in the initial four districts during the second round (Figure 4). Good coverage was sustained in Simdega, and the other districts showed an increase in coverage during the second round. This increase inspired confidence among program managers that high coverage of IDA is feasible when planning, training, and social mobilization are conducted according to the newly developed guidelines.

## APPROACHES AND STRATEGIES USED FOR TRIPLE-DRUG IDA ROLLOUT IN INDIA

Key stakeholders, including the central government, state government, district, and block-level health officials, adopted tailored approaches to rolling out IDA. Efforts focused on advocacy, information, education, and communication (IEC) efforts to build awareness, social mobilization, deployment of more teams to administer drugs, and supervision and monitoring. The partners’ role in filling gaps worked as a catalytic support for ensuring effective implementation of the rounds of IDA MDA. Strategies and approaches that contributed to the successful implementation of IDA rollout in India are described in more detail as follows.

### Strong political will.

As an immediate outcome of advocacy efforts during the Global Alliance to Eliminate Lymphatic Filariasis meeting in June 2018, J. P. Nadda, former Union Minister, chaired a review meeting with the state health ministers and principal secretaries of health from seven endemic states to prioritize LF elimination. Subsequently, the state health ministers of Bihar and Maharashtra undertook review meetings of their respective state LF elimination programs. A total of 17 members of parliament and members of the legislative assembly actively supported IDA rollout in the initial pilot districts.

### Strong bureaucratic ownership.

Sustained involvement of bureaucratic leadership at the national and state levels ensured that the district level prioritized IDA rollout as an important public health priority. This was achieved through well-coordinated efforts under the leadership of NVBDCP and strong coordination with neglected tropical disease partners, state officials, and districts officials. Joint action plans were developed and agreed upon to effectively plan and implement activities before, during, and after MDA rounds.

### Task forces and guidelines.

The state, district, and block task forces were composed of staff from the Ministry of Health and Family Welfare, other government departments, and partners. Existing training processes were modified according to IDA guidelines and were simplified in Hindi, the local language. Social mobilization and interpersonal communication sessions for frontline workers were incorporated in all trainings, including the state-level training of trainers, district-level training of trainers, and drug administrator training. The number of days allotted to conduct the MDA campaign were increased to ensure complete coverage and compliance of the target population.

### Training of health workers and drug administrators.

Enhanced training of medical officers, frontline workers, supervisors, and drug administrators before the MDA rounds covered aspects such as the proper use of dose poles, clarity on IDA dosages, and training on key messages for community members on the benefits of IDA. Drug administrators were well equipped with newly designed IEC tools to engage with the community effectively.

### Innovative drug administration strategies.

**Figure 5.**
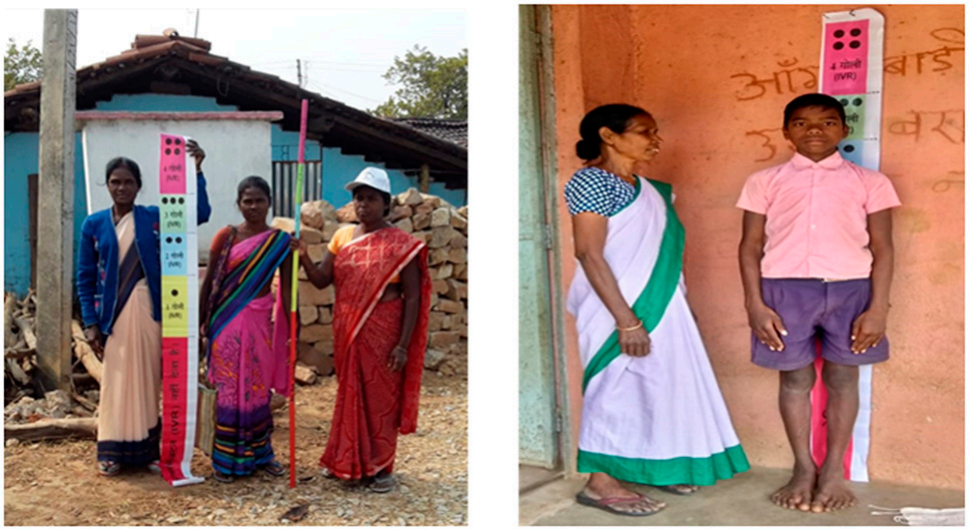
Use of dose poles for ivermectin with DEC and albendazole (IDA) administration.

The MDA campaigns used innovative approaches that focused on supervised administration at single-day booths in schools (after midday meals), Anganwadi centers, and health facilities. These innovative strategies were implemented in combination with door-to-door administration and mop-up rounds. Additionally, finger marking using marker pens was adopted to indicate who had already received IDA, including school children covered at booths. NVBDCP, in close coordination with partners, developed an innovative color-marked dose pole to determine the required dosage of ivermectin based on the height of the individual, according to WHO guidelines (Figure 5). Finger marking was initiated after drug administration and upon receiving IEC messaging.

### Microplanning.

Supervisors and drug administrators received training to improve the quality of microplanning for rural and urban areas. Good-quality microplans were of great help in reducing missed areas. Training at each level emphasized the importance of good-quality microplanning for effective implementation.

### IEC and social mobilization.

IEC materials including billboards, banners, posters, wall paintings, brochures, handbills, and newspaper advertisements were developed and shared in sufficient numbers well in advance of IDA rounds in the select districts. In addition to media engagement and crisis communications, branding and brand ambassadors, digital communications, and high profile launch events (detailed below), the following initiatives for community mobilization and awareness generation were also adopted:

• Community mobilizers were deployed at the block level to facilitate mobilization.
• All existing platforms available in the districts were activated, such as schools, leaders of panchayat (local self-governance units), local leaders, and women’s collectives.
• Advance notice to communities was ensured. Community members received information about possible side effects and reasons for occurrence and how to report any adverse effects to community health workers, Anganwadi or health workers at booths, or the nearest subcenter or primary health center.
• In coordination with the education department, a special school awareness program called Bhag Filaria Bhag (“go filaria, go”) was initiated in all schools 15 days before MDA rounds began. Essay and drawing competitions were also organized in schools with winners receiving certificates of appreciation.
• Sensitization of block-level public representatives and faith-based organizations was conducted.
• Awareness rallies and wall writing also contributed to enhancing community reach and awareness.

### Media engagement and crisis communications.

Media workshops were organized 2 days before the launch ceremony of IDA to disseminate key messages on the benefits of IDA and promote the dates of IDA rounds through print, broadcast, and digital channels. Crisis communication plans were developed to engage with the media in case of any serious adverse event to ensure a high level of trust among community members and preparedness to address misinformation.

### Branding of LF program.

**Figure 6.**
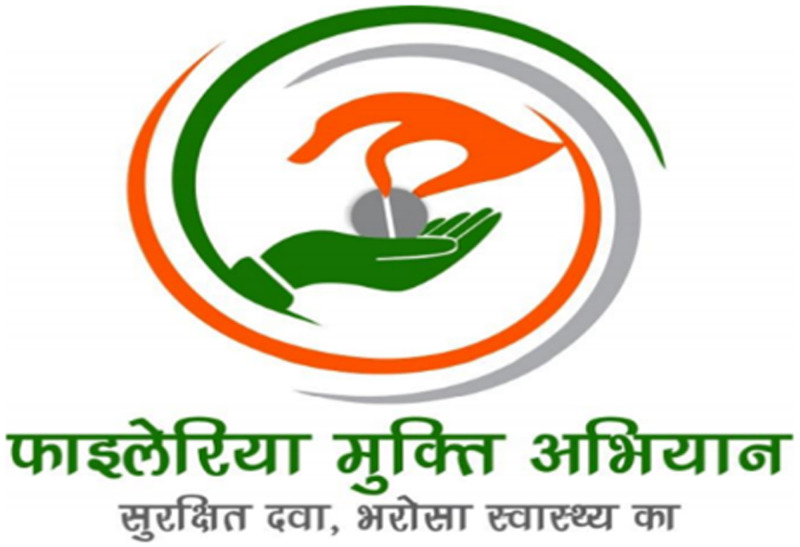
New logo for the program. Note: The bold line below the design says “Filaria Free Campaign” with the tagline: “Safe drug, assurance for better health.”

A logo and tagline were developed to enhance brand recognition and community acceptance for the program. The logo includes two hands—the first hand signifies the health worker offering the IDA treatment and the second hand indicates the community member accepting the treatment. The tagline is “safe drug, assurance for better health” (Figure 6).

### Brand ambassador for LF elimination.

State governments were supported to officially recruit a popular actor and youth icon as the brand ambassador for LF elimination in the state of Maharashtra where IDA was to be piloted in Nagpur District, which includes over 2 million people in urban areas. The actor’s engagement to support IDA introduction ranged from developing audio and video messages, authoring media articles, and leveraging his vast social media network to popularize the importance of addressing LF as a public health problem and acceptance of IDA by the community.

### Digital communication through social media.

Facebook and Twitter campaigns (#IndiaWillEndLF) promoting the elimination of LF were used to amplify the digital reach of communication messages about triple-drug MDA rounds. Social media messages were customized and shared through WhatsApp groups and widely disseminated through the networks of state, district, and community government officials, technical experts, and frontline workers.

### High-visibility IDA launch in pilot districts.

To increase the visibility of IDA rollout, launch events included the participation of elected representatives, popular celebrities, technical experts, state- and district-level officials (at the block level and the district level, respectively), and the media.

### Enhanced monitoring and supervision.

Effective supervision with one supervisor for every five booths or teams and a system of daily reporting from the supervisor to the block level by each evening was implemented. Simple formats facilitated daily reporting from booths to supervisors, supervisors to block levels, blocks to district headquarters, and districts to state headquarters. Rapid response teams headed by a medical officer with mobility support were identified in each block. In blocks with larger populations, two rapid response teams were identified. All adverse events were immediately attended to, while those with serious adverse events were hospitalized and adequately managed.

### Post-MDA assessment and evaluation.

Designated medical colleges carried out the post-MDA assessment according to established guidelines within 15 days after completion of MDA. A team formed by the state- and the district-level officials and one representative from NVBDCP conducted the post-MDA evaluation. Partner organizations including the WHO and Project Concern International also conducted independent small sample size data collection for post-MDA monitoring. Low coverage areas were followed up with additional “mop up” activities to cover the left-out populations.

### Support by partner agencies.

Partner support was vital for the successful implementation of IDA. WHO provided country support by developing standardized technical guidelines and training materials, operational research, and logistical support for drugs. Project Concern International supported social mobilization in selected states; PATH provided technical support for developing guidelines and tools; and Global Health Strategies undertook national-, state-, and district-level advocacy and communications.

## CRITICAL CHALLENGES TO OVERCOME FOR THE SUCCESSFUL INTRODUCTION OF IDA

• The Drug Controller General of India had not approved ivermectin for human use in India, so the technical committee decided to seek approval, which required additional time and effort by NVBDCP to secure necessary approvals.
• The supply of drugs was delayed due to administrative challenges and dates of MDA had to be revised.
• During IDA rollout in Bihar, there was a local strike of accredited social health activists (ASHA or community health workers instituted by the Ministry of Health and Family Welfare). Alternatively, other cadres such as Anganwadi workers were involved and booths were established in Anganwadi centers and schools. MDA was initiated but was interrupted after 2 weeks when Anganwadi workers also joined the strike.
• Because MDA had never been undertaken in urban parts of Nagpur district or Simdega district, a lot of preparatory work was required, including mapping, developing microplans, and setting up systems as well as work to promote community awareness and conduct outreach activities.
• In Varanasi district, IDA had to be conducted in February 2019. However, the Kumbh Mela (an elaborate religious gathering of pilgrims) took place at the same time and most of the government machinery was deployed for the event. Postponing IDA was not an option because general elections were scheduled for April 2019, which would have further delayed the rollout of IDA. Elections impact the overall reach to community members because health staff are deployed for election duties.
• The delay in fabricating and dispatching dose poles from the central level led to the recommendation that dose poles should be developed at the local level. This helped prevent implementation delays.
• Because of low coverage rates, the “mop-up” activity, which normally took from 7 to 10 days, to reach people who were missed during the initial efforts was extended to 2 or 3 weeks in some areas, which led to additional time and effort by drug administrators (without increasing the budget).

## LESSONS LEARNED

• Lessons learned from India’s Polio Eradication Program were optimally applied for the successful introduction of IDA. Polio eradication principles were followed in all aspects of IDA rollout, including microplanning, training, social mobilization, engaging with the media, developing a logo and tagline, recruiting a brand ambassador, using finger marking and house marking, monitoring, strengthening data quality, and conducting program reviews at all levels. State and district administrators and partners easily understood the connection and were successful in their efforts.
• For securing approval of the new drug combination, past experiences from introducing the Japanese encephalitis vaccination in campaign in India were helpful. Getting the right stakeholders at the table was a crucial step learned in the previous decade. Being cautious and honest about the possibilities of adverse events after the introduction of a new vaccine/drug and ensuring awareness of a strong rapid response mechanism inspired confidence among Indian stakeholders in regard to introducing and scaling up IDA.
• Districts with a large urban population required detailed mapping of areas to be covered and advance planning to engage all existing resources. Engaging with municipal corporations yielded good results. Urban health units need to be sensitized to increase ownership of the campaign.
• Convincing apparently healthy and asymptomatic adults to consume eight tablets at a time is a challenge. There is a need for targeted and enhanced communications and social mobilization to effectively reach out to communities.
• Migrant people, particularly labor-intensive populations from LF endemic states, move frequently in search of work and livelihood. This demands a convergent approach to engage allied departments to cover these migratory populations.
• Greater involvement of local-level panchayat and functionaries enhances efforts to reach out to communities. Advance preparation for advocacy, IEC, and social mobilization efforts is greatly beneficial.
• Increased number of booths for administering drugs, along with household visits, helped increase program reach. Household members who were absent during door-to-door campaigns were visited subsequently during mop-up activities to ensure drug consumption. This helped redesign and improved microplans for further IDA rounds.
• A daily monitoring system, reporting of adverse events, and arranging for the treatment of those with adverse effects should be in place for the duration of MDA rounds and until 4–5 days after completion of the rounds.

## ROLE OF IDA IN REACHING PROGRAM GOALS

Because India has a huge population to be covered during MDA campaigns, the greatest need is domestic procurement of ivermectin to scale up IDA in persistently endemic implementation units. Ultimately, 50 million treatments donated by the Mectizan Donation Program per year will not be sufficient for the 430 million people who can benefit by receiving the more effective triple-drug IDA regimen to accelerate elimination goals in India. Ensuring a good-quality drug supply from domestic manufacturers is an area of concern for India.

To improve program implementation and targeted community compliance during MDA rounds, there is a need to reduce the size of implementation units. Focusing on blocks as the implementing unit and evaluating transmission at the block level can help in identifying blocks with ongoing transmission versus blocks that have achieved transmission interruption. Targeting and scaling up IDA in identified blocks in known endemic districts can play a major role in curbing ongoing transmission and enabling other blocks to move to post-MDA surveillance activities.

## MDA IMPLEMENTATION SUPPORT BEYOND IDA

**Figure 7.**
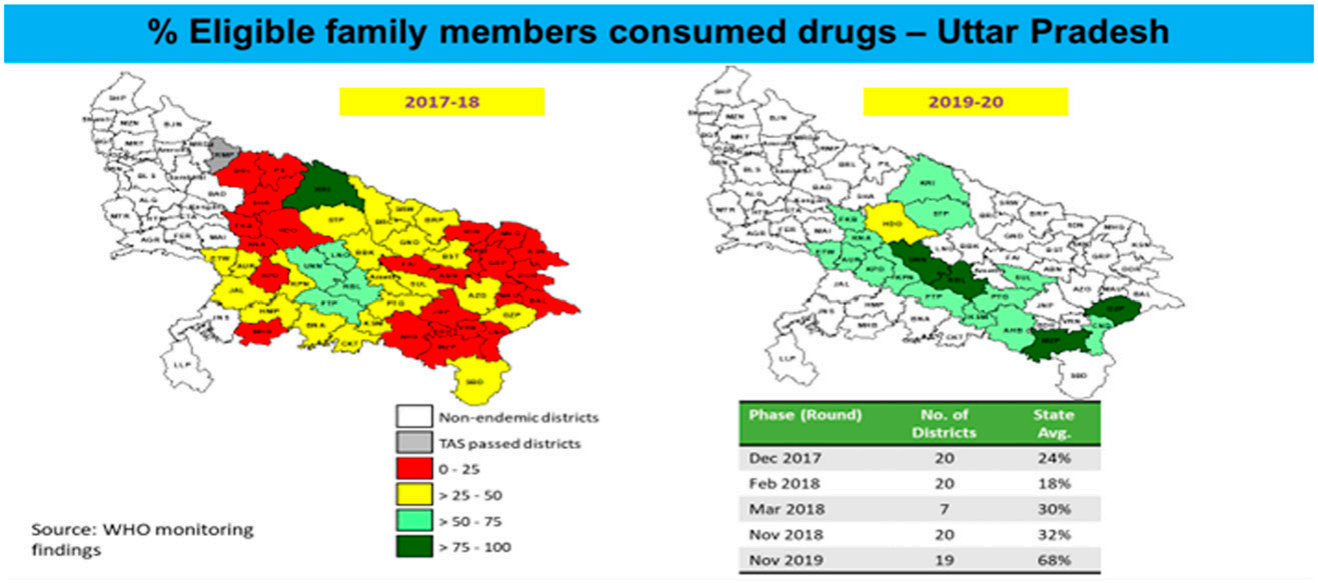
Improvement in coverage over time, Uttar Pradesh, India. Source: WHO India Country Office LF MDA field monitoring data.

**Figure 8.**
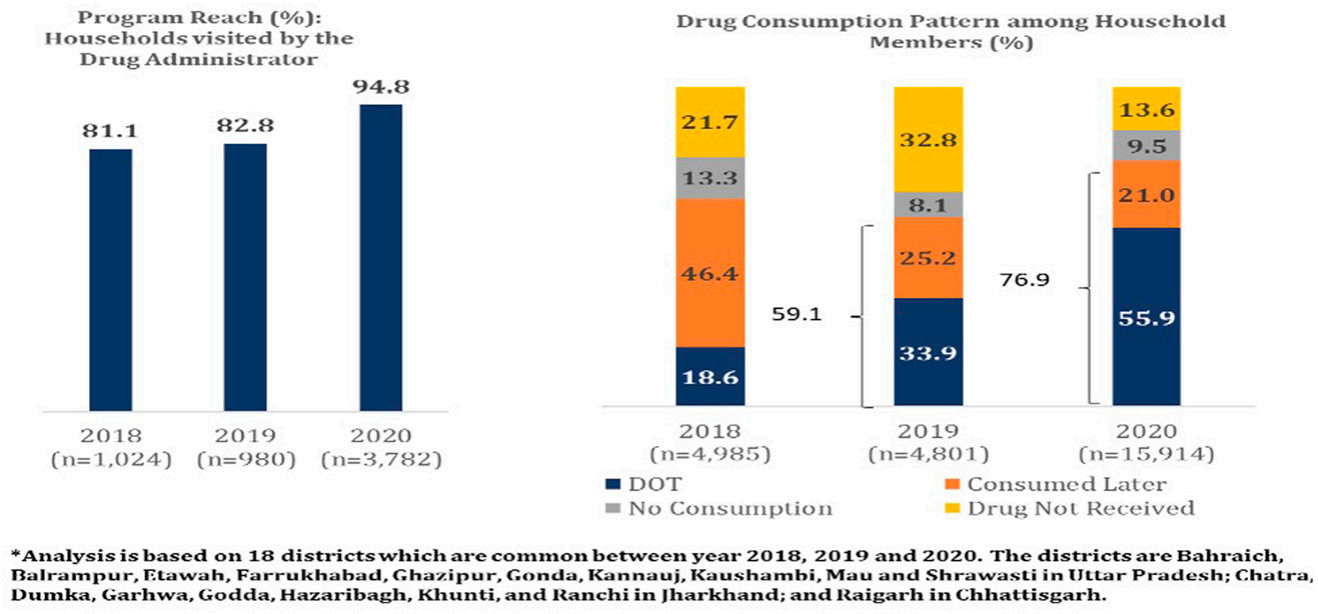
Comparative data over 3 years for program reach and drug consumption. Source: Social Mobilization field study by Project Concern International.

The Government of India received additional support from partners to strengthen not only the introduction of triple-drug IDA but also double-drug DA campaigns. There was a significant improvement in overall program performance from those districts as well (Figure 7). The proportion of directly observed therapy for drug consumption increased from 18.6% to 55.9% over a period of 3 years between 2018 and 2020 (Figure 8). This was due to a change in training for drug administrators (with a focus on directly observed therapy), enhanced prioritization by states and districts, and better social mobilization at the last mile.

While reviewing subnational data, significant improvements were observed at the district level. An example of district-level data for the state of Uttar Pradesh is shown in Figure 7.

The current strategy is to strengthen the program in all geographies, irrespective of whether MDAs have been carried out using double-drug or triple-drug regimens (Figure 8).

## THE ROAD AHEAD FOR LF ELIMINATION

The following measures will assist in the rapid scale-up of IDA in India and further improve compliance among communities to sustain reductions in LF transmission during the post-IDA period:

• Public funding and budget allocation for triple-drug MDA rounds should be revised as rates that were fixed a decade ago by the Ministry of Health are insufficient for achieving desired results. This will ensure orientation of health staff, involvement of allied health department, and the alignment of IEC, and social mobilization to the needs of each area.
• Urban areas need more attention as the health system doesn’t have sufficient govt./accountable drug administrators/supervisors and there is a lack of updated population registery for all individuals. Officer goers, shopkeepers, and private schools will need extra efforts for optimal coverage. Microplanning will need adjustments to administer drugs at institutions and to plan revisits when most residents are expected to be at home. Resident welfare associations and Multistorey building management will have to cooperate with drug administrators for better coverage. Improved and innovative social media usage will help in awareness generation and acceptance of drugs.
• IDA should be given to migrant populations of daily wage workers if they are in large cluster groups from LF endemic areas working in various industries such as mines and factories located in other mosquito-endemic geographies.
• Strong monitoring and evaluation system will be required to measure the success and provide evidence for corrective action.

Proper documentation will be required for WHO validation of elimination in the future.

## Supplemental Material


Supplemental materials
