# Supplementary material for: Introduction of Triple-Drug Therapy for Accelerating Lymphatic Filariasis Elimination in India: Lessons Learned
Source: Am J Trop Med Hyg. 2022 Mar 15;106(5 Suppl):29–38. doi: 10.4269/ajtmh.21-0964 (PMC9154644; doi:10.4269/ajtmh.21-0964)

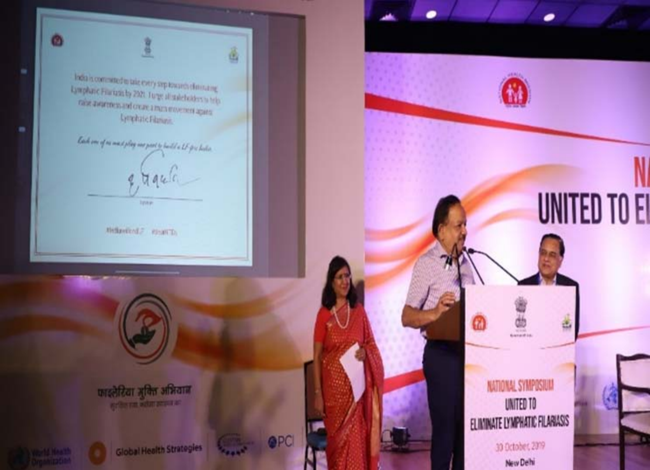
UNITED TO EL
फाइलेरिया मुक्ति अभियान
World Health Organization
Global Health Strategies
PCI
NATIONAL SYMPOSIUM
UNITED TO
ELIMINATE LYMPHATIC FILARIASIS
30 October, 2019
New Delhi

MDA से सम्बंधित आवश्यक गतिविधियाँ :

- गाँव के मुखिया की उपस्थिति में MDA से पूर्व ग्राम सभा कर, लोगों को फाइलेरिया एवं इससे बचाव की जानकारी देना तथा निर्धारित तिथि को चिन्हित बूथ पर दवा के सेवन हेतु प्रेरित करें।
- लक्षित आबादी के अनुसार दवाइयों की उपलब्धता सुनिश्चित करें।
- घर के सभी सदस्यों को उनकी उम्र के अनुसार गोलियाँ दें तथा सुनिश्चित करें कि सभी लोग आपके सामने ही दवा खाएँ।
- अपने पास पर्याप्त मात्रा में दवा, परिवार रजिस्टर, व फ्लैश कार्ड्स रखें।
- अगर दवा खाने के बाद किसी तरह की परेशानी हो तो नज़दीकी स्वास्थ्य केंद्र जाने की सलाह दें एवं अविलम्ब त्वरित कार्यदल को सूचित करें।
- सभी MDA बूथ पर प्रचार प्रसार सामग्री का प्रदर्शन करें।
- अपने क्षेत्र में निर्धारित तिथि से 2 दिन पूर्व से ही माइकिंग द्वारा प्रचार-प्रसार सुनिश्चित करें।

एमडीए की खुराक दवा तालिका

ऊंचाई (सेंटीमीटर में)	आइवर्मेक्टिन (एक गोली 3 मिलीग्राम)
89 से.मी. से कम	नहीं देना है
90-119 से.मी.	01 गोली
120-140 से.मी.	02 गोली
141-158 से.मी.	03 गोली
159 से.मी. से अधिक	04 गोली

उम्र	डी.ई.सी (एक गोली 100 मिलीग्राम)	अल्बेन्डाजोल (एक गोली 400 मिलीग्राम)
2 साल से कम	नहीं देना है	नहीं देना है
2-5 साल	1 गोली	1 गोली
6-14 साल	2 गोलियाँ	1 गोली
15 साल तथा उससे बड़े	3 गोलियाँ	1 गोली

दवा प्रशासक मार्गदर्शिका

फाइलेरिया मुक्ति अभियान

"सुरक्षित दवा, बढ़िया स्वास्थ्य का"

फाइलेरिया क्या है?

फाइलेरिया मच्छर के काटने से होने वाला एक संक्रामक रोग है जिसे सामान्यतः हाथीपाँव के नाम से जाना जाता है।

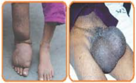

फाइलेरिया के लक्षण

- ✓ पैरों व हाथों में सूजन (हाथीपाँव) और अण्डकोष का सूजन (हाइड्रोसिल)।
- ✓ किसी भी व्यक्ति को संक्रमण के पश्चात बीमारी होने में 5 से 15 वर्ष तक लग सकते हैं।

फाइलेरिया मुक्ति अभियान

"सुरक्षित दवा, बढ़िया स्वास्थ्य का"

[illegible]-11 जनवरी 2019

फाइलेरिया क्या है ?

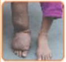

- फाइलेरिया मच्छर के काटने से होने वाला एक संक्रामक रोग है।
- व्यक्ति किसी भी उम्र में फाइलेरिया से संक्रमित हो सकता है।
- फाइलेरिया के लक्षण हाथ और पैर की सूजन (हाथीपाँव) व अण्डकोष का सूजन (हाइड्रोसिल) है।

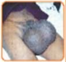

फाइलेरिया का रोकथाम / निवारण

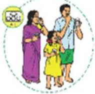

आई.डी.ए - एम.डी.ए. के दौरान [illegible] द्वारा मुफ्त में दी जाने वाली फाइलेरिया रोधी दवा खाएं (आइवर्मेक्टिन, डी.ई.सी. व अल्बेन्डाजोल)

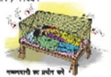

मच्छरदानी का प्रयोग करें

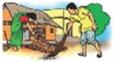

अपने आस-पास जल-जमाव न होने दें

दवा खाने के फायदे क्या हैं ?

- यह दवा फाइलेरिया के रोगाणु को मार देती है और व्यक्ति हाथीपाँव व हाइड्रोसील जैसी बीमारी से बचाने में मदद करती है।

याद रखें

- दो साल से कम उम्र के बच्चे, गर्भवती महिलाओं और गंभीर रोग से पीड़ित व्यक्तियों के अलावा यह दवा सभी को खानी है।
- यह दवा उम्र एवं लम्बाई के आधार पर दी जाएगी।
- यह दवा खाली पेट नहीं खानी है।
- दवा स्वास्थ्य कर्मी के सामने ही खाना [illegible] है।
- दवा सेवन के बाद कभी-कभी चक्कर, शरीर में दर्द, बुखार, उल्टी तथा बदन पर चकत्ते एवं खुजली जैसी मामूली प्रतिक्रियाएँ देखने को मिलती हैं, जो फाइलेरिया के रोगाणु के नष्ट होने को दर्शाती हैं।
- घबराएं नहीं आवश्यकता पर ये लक्षण स्वतः ही ठीक हो जाते हैं।

भाग फाइलेरिया भाग

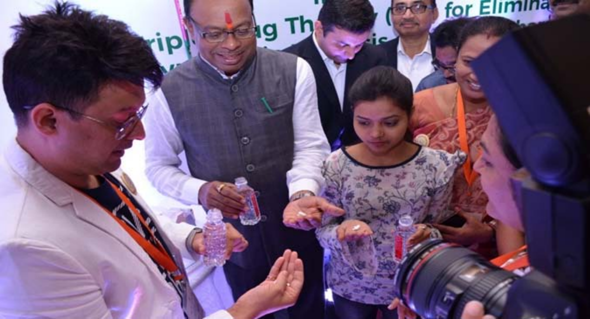

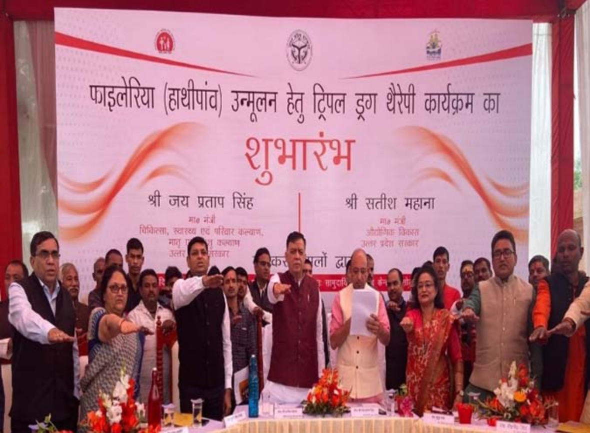
फाइलेरिया (हाथीपांव) उन्मूलन हेतु ट्रिपल ड्रग थैरेपी कार्यक्रम का
शुभारंभ
श्री जय प्रताप सिंह
मा० मंत्री
चिकित्सा, स्वास्थ्य एवं परिवार कल्याण,
श्री सतीश महाना
मा० मंत्री
औद्योगिक विकास
उत्तर प्रदेश सरकार

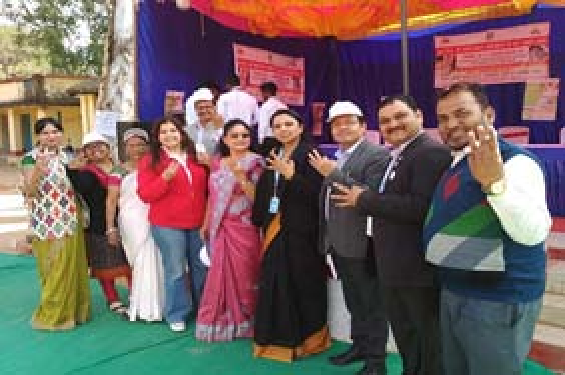

Supplement: Supplementary file 1 [file tpmd210964.SD1.pdf]
